# The Polar *Legionella* Icm/Dot T4SS Establishes Distinct Contact Sites with the Pathogen Vacuole Membrane

**DOI:** 10.1128/mBio.02180-21

**Published:** 2021-10-12

**Authors:** Désirée Böck, Dario Hüsler, Bernhard Steiner, João M. Medeiros, Amanda Welin, Katarzyna A. Radomska, Wolf-Dietrich Hardt, Martin Pilhofer, Hubert Hilbi

**Affiliations:** a Institute of Molecular Biology and Biophysics, Department of Biology, ETH Zürich, Zürich, Switzerland; b Institute of Medical Microbiology, Faculty of Medicine, University of Zürich, Zürich, Switzerland; c Institute of Microbiology, Department of Biology, ETH Zürich, Zürich, Switzerland; Institut Pasteur

**Keywords:** bacterial pathogenesis, cryo-electron tomography, Legionnaires’ disease, type IV secretion system, pathogen vacuole, *Legionella pneumophila*

## Abstract

Legionella pneumophila, the causative agent of Legionnaires’ disease, is a facultative intracellular pathogen that survives inside phagocytic host cells by establishing a protected replication niche, termed the “*Legionella*-containing vacuole” (LCV). To form an LCV and subvert pivotal host pathways, L. pneumophila employs a type IV secretion system (T4SS), which translocates more than 300 different effector proteins into the host cell. The L. pneumophila T4SS complex has been shown to span the bacterial cell envelope at the bacterial poles. However, the interactions between the T4SS and the LCV membrane are not understood. Using cryo-focused ion beam milling, cryo-electron tomography, and confocal laser scanning fluorescence microscopy, we show that up to half of the intravacuolar L. pneumophila bacteria tether their cell pole to the LCV membrane. Tethering coincides with the presence and function of T4SSs and likely promotes the establishment of distinct contact sites between T4SSs and the LCV membrane. Contact sites are characterized by indentations in the limiting LCV membrane and localize juxtaposed to T4SS machineries. The data are in agreement with the notion that effector translocation occurs by close membrane contact rather than by an extended pilus. Our findings provide novel insights into the interactions of the L. pneumophila T4SS with the LCV membrane *in situ*.

## INTRODUCTION

Legionella pneumophila is a Gram-negative, opportunistic pathogen and the main cause of a life-threatening pneumonia in humans, known as Legionnaires’ disease (1). L. pneumophila is well-adapted to living in complex multispecies biofilm communities, where it persists and replicates intracellularly in a variety of protozoa, such as Acanthamoeba castellanii (2–4). Under laboratory conditions, L. pneumophila also replicates in the genetically tractable social amoeba Dictyostelium discoideum (5). To survive inside host cells, L. pneumophila establishes a unique, membrane-bound compartment, the replication-permissive “*Legionella*-containing vacuole” (LCV) (6, 7). LCVs avoid the fusion with bactericidal lysosomes but vividly communicate with host vesicle trafficking along the endocytic, secretory, and retrograde pathways (8) and ultimately associate with and exploit the dynamics of the endoplasmic reticulum (ER) (9). Formation and maintenance of the LCV are key for pathogenesis and are strictly dependent on the Icm/Dot (intracellular multiplication/defective in organelle trafficking) type IV secretion system (T4SS), a bacterial conjugation apparatus (10, 11). The Icm/Dot T4SS constitutes an essential virulence factor, since bacteria lacking the secretion system are not pathogenic and cannot replicate within host cells (12). The T4SS injects more than 300 different putative effector proteins into the host cell cytosol, which interfere with various cellular processes, e.g., vesicle trafficking, actin dynamics, or signal transduction (13–15).

Polar secretion of substrates is crucial for the establishment of the LCV and is attributed to the polar localization of the T4SS (16–18). In fact, upon translocation, many effector proteins are retained at the cytoplasmic face of the LCV membrane adjacent to the bacterial cell poles (19–23). Two Icm/Dot proteins, DotU and IcmF, target the T4SS to the cell poles independently of the other Icm/Dot components (17). DotU and IcmF are homologs of TssL and TssM, which are components of the type VI secretion system (T6SS) trans-envelope complex (24–26). The importance of the cell poles as assembly sites for secretion systems has been demonstrated for different secretion systems (type II, III, IV, or VI) and various bacterial pathogens (27–31). Hence, the polar localization of secretion systems seems to be a conserved trait, providing a fitness advantage for some intracellular pathogens, including L. pneumophila.

T4SSs are specialized macromolecular delivery machines that can be classified as type IVA (T4ASS) or IVB (T4BSS), depending on whether their structural components resemble the VirB/D4 complex of Agrobacterium tumefaciens or the conjugation transfer systems of IncI plasmids, respectively (32–35). The Icm/Dot T4BSS of L. pneumophila comprises 27 proteins that adopt a “Wi-Fi”-like structure and span the bacterial cell envelope (34, 36, 37). Conformational changes in the cytoplasmic portion of the T4SS, triggered by the ATPases DotO and DotB, result in the opening of a channel in the bacterial inner membrane, enabling transport of cognate substrates across the cytoplasmic membrane (18). A continuous secretion channel bridging both the bacterial inner and outer membranes has not yet been identified.

Due to the homology between the Icm/Dot T4SS and an ancestral IncI conjugation system (38), a pilus-like structure might be involved in substrate translocation into the host cytoplasm. Yet, a pilus associated with the Icm/Dot T4SS has never been observed in L. pneumophila, and bioinformatic analyses indicate that the T4SS lacks the type IV pilus gene cluster (39). Moreover, recently published high-resolution subtomogram averages of the Icm/Dot T4SS in L. pneumophila grown in broth also did not identify a pilus structure (18, 36, 37, 40). Hence, it is still unclear how the T4SS interacts and establishes contact with the LCV membrane in order to transport effector proteins across the bacterial inner and outer membranes and the LCV into the host cytosol.

Here, we applied a combined approach of cryo-focused ion beam (cryoFIB) milling, cryo-scanning electron microscopy (cryoSEM), cryo-electron tomography (cryoET), and confocal laser scanning fluorescence microscopy (CLSM) to study the interactions between the L. pneumophila Icm/Dot T4SS and host membranes. Our data reveal that intracellular L. pneumophila tethers its cell pole to the LCV membrane to establish distinct contact sites between the T4SS and the pathogen vacuole. The contact sites are characterized by indentations in the LCV membrane toward the T4SS and are specific for functional secretion machineries located juxtaposed to the limiting pathogen vacuole membrane.

## RESULTS AND DISCUSSION

### L. pneumophila tethers its cell pole to the LCV membrane.

**FIG 1:**
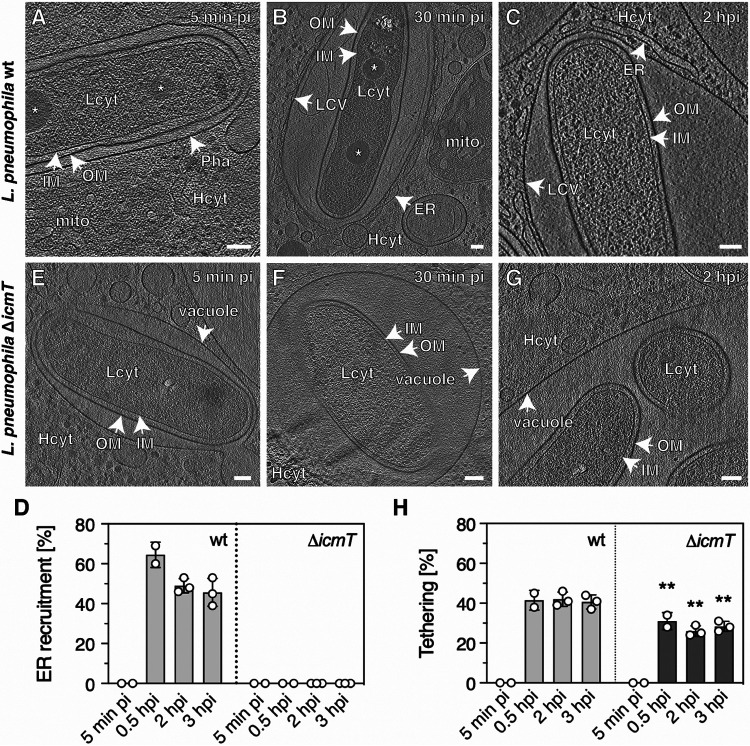
L. pneumophila wild-type tethers its cell pole to the LCV membrane at early and late infection stages. A. castellanii amoebae were infected with L. pneumophila wild-type (A to C) or the Δ*icmT* mutant (E to G) for the time indicated. Representative 2D images of cryotomograms (12-nm tomographic slices) of L. pneumophila wild-type residing in a tight phagosome at very early infection stages (5 min pi, *n*^vacuoles^ = 10) (A) or in a mature, ER-decorated LCV (30 min pi, *n*^LCVs^ = 29; 2 h pi, *n*^LCVs^ = 38) (B, C), with the cell pole tethered to the LCV membrane (∼41% on average over time, *n*^cell poles^ = 202). (D) Quantification of data shown in panels A to C and E to G. (E to G) T4SS-defective Δ*icmT* mutant bacteria oriented their poles to the vacuole membrane less frequently (∼28% on average over time, *n*^cell poles^ = 45). (H) Quantification of data shown in panels A to C and E to G. Data in panels D and H are represented as mean ± standard deviation (SD) from at least two independent infection experiments (one-way ANOVA test; **, *P *< 0.01). OM, outer membrane; IM, inner membrane; Pha, phagosome; LCV, LCV membrane; Lcyt, L. pneumophila cytoplasm; Hcyt, host cell cytoplasm; ER, endoplasmic reticulum; mito, mitochondrion; asterisk, storage granule; Scale bars, 100 nm.

To understand ultrastructural features of the Icm/Dot T4SS during effector translocation, we infected two amoeba hosts—A. castellanii and D. discoideum—with L. pneumophila JR32 (a derivative of wild-type strain L. pneumophila Philadelphia-1, hereafter referred to as “wild-type”) and analyzed intracellular bacteria using cryoFIB milling, cryoSEM, and cryoET. CryoFIB milling can reduce the thickness of voluminous biological samples, such as eukaryotic cells to a few hundred nanometers, allowing for subsequent high-resolution cryoET imaging of cell architecture as well as intracellular pathogens (41, 42). Cryotomograms showed that shortly upon uptake into A. castellanii, L. pneumophila resided in a tight phagosome (Fig. 1A, 5 min postinfection [pi]), which was modified into the specialized and more spacious LCV as the infection progressed (Fig. 1B and C, 30 min pi and 2 h pi). A distinguishing feature between phagosomes (Fig. 1A) and mature LCVs (Fig. 1B and C) is that the latter are decorated with the endoplasmic reticulum (ER). Recruitment of the ER to the LCV is a hallmark of this replication-permissive pathogen compartment and is mediated by effector proteins such as SidC (43, 44). While ER recruitment was not yet initiated at 5 min pi (Fig. 1D; *n*^vacuoles^ = 10), it became highly prominent at 30 min pi (65% of LCVs, *n*^LCVs^ = 29), 2 h pi (49%, *n*^LCVs^ = 38), and 3 h pi (46%, *n*^LCVs^ = 38) in A. castellanii. Comparable results were obtained when D. discoideum was used as a host cell (see Fig. S1A to C in the supplemental material; 64% of LCVs at 0.5 h pi, *n*^LCVs^ = 18; 43% of LCVs at 2 h pi, *n*^LCVs^ = 23; 41% of LCVs at 3 h pi, *n*^LCVs^ = 36). Data from both host cells therefore confirm that our infection protocols allow for the analysis of functional Icm/Dot T4SSs, which are required for LCV maturation and ER acquisition.

10.1128/mBio.02180-21.1 FIG S1 L. pneumophila wild-type tethers its cell pole to the LCV membrane in D. discoideum. Download FIG S1, PDF file, 1.0 MB. Copyright © 2021 Böck et al. 2021 Böck et al. https://creativecommons.org/licenses/by/4.0/ This content is distributed under the terms of the Creative Commons Attribution 4.0 International license.

Interestingly, cryoET data also revealed that up to half of the bacterial poles remained in close proximity (<35 nm) to the LCV membrane. The poles indeed localized closer to the vacuole membrane than the lateral side of the bacteria throughout pathogen vacuole expansion and maturation (Fig. 1B and C; see also Fig. S1; *n*^cell-poles^ = 202). CryoSEM imaging of lamellae indicated similar associations of the bacterial cell pole and the LCV membrane (see Fig. S2 in the supplemental material; *n*^vacuoles^ = 223).

10.1128/mBio.02180-21.2 FIG S2 L. pneumophila wild-type tethers its cell poles to the LCV membrane. Download FIG S2, PDF file, 0.9 MB. Copyright © 2021 Böck et al. 2021 Böck et al. https://creativecommons.org/licenses/by/4.0/ This content is distributed under the terms of the Creative Commons Attribution 4.0 International license.

To analyze whether the orientation of the bacterial poles toward the LCV membrane was dependent on the presence and/or function of the T4SS, we performed identical infection experiments with the L. pneumophila Δ*icmT* mutant strain, which lacks IcmT, an inner membrane component of the Icm/Dot T4SS. The Δ*icmT* mutant produces a defective T4SS, does not form a replication-permissive LCV, and shows severe defects in bacterial conjugation, macrophage killing, and survival in amoebae (10, 45, 46). Cryotomograms of A. castellanii (Fig. 1E to G; *n* = 18) and D. discoideum (Fig. S1C to E; *n* = 13) infected with the L. pneumophila Δ*icmT* strain revealed that these bacteria-containing vacuoles were never decorated with ER (Fig. 1D), confirming the functional defect of the T4SS. Compared to the wild-type strain, cell pole tethering (defined as <35-nm spacing between the bacterial outer membrane and the LCV membrane in a spacious vacuole) was observed at a significantly lower frequency for the Δ*icmT* mutant bacteria in A. castellanii (Fig. 1H) and D. discoideum (Fig. S1). Within our experimental time frame (3 h), we observed an ∼1.5-fold reduction of cell pole tethering between the L. pneumophila wild-type and Δ*icmT* strain (on average 41% versus 28%; *n*^cell poles-mutant^ = 45 and *n*^cell poles-wild-type^ = 202). Taken together, these findings suggest that structural components of the Icm/Dot T4SS and/or its function (effector translocation) play a role in tethering the bacterial cell pole to the LCV membrane.

### The Icm/Dot T4SS localizes to the poles of intracellular L. pneumophila.

The Icm/Dot T4SS has been identified at bacterial cell poles in the L. pneumophila strain Lp02 grown in broth (16, 36, 37, 40). We hypothesized that tethering of the bacterial cell pole to the LCV membrane might be a consequence of the polar localization of the T4SS. Accordingly, we analyzed by cryoET the localization of T4SSs in the L. pneumophila strain JR32 used in this study (strain JR32 and strain Lp02 are both Philadelphia-1 derivatives). Cryotomograms confirmed the polar localization of T4SSs in L. pneumophila JR32 as well as in Δ*icmT* mutant bacteria (see Fig. S3 in the supplemental material; average of ∼3 T4SSs/cell pole, range of 1 to 6 T4SSs/cell pole, *n*^cell poles^ = 47). While lateral T4SSs were identified at low frequency (∼1.6 per cell) in a previous study (18), we did not observe nonpolar T4SSs in JR32 or the Δ*icmT* strain. This discrepancy could, however, be due to different strains and growth conditions used in the previous study (solid medium) and our study (liquid medium). Among the JR32 and Δ*icmT* mutant cell poles analyzed, 17% harbored one and 66% harbored multiple polar T4SSs (Fig. S3; *n*^cell poles JR32^ = 28, *n*^cell poles Δ^*^icmT^* = 19). In conclusion, despite the functional defect of the L. pneumophila Δ*icmT* strain (Fig. 1E to G; see also Fig. S1) (10, 45, 46), the mutant bacteria retain the subunits required to assemble the characteristic “Wi-Fi-like” structure of the T4SS. Moreover, our data confirm previous reports on the polar localization and copy number of T4SSs in L. pneumophila (16, 18, 36, 37, 40).

10.1128/mBio.02180-21.3 FIG S3 L. pneumophila Icm/Dot T4SS is located at bacterial cell poles. Download FIG S3, PDF file, 0.9 MB. Copyright © 2021 Böck et al. 2021 Böck et al. https://creativecommons.org/licenses/by/4.0/ This content is distributed under the terms of the Creative Commons Attribution 4.0 International license.

We also assessed the localization of the Icm/Dot T4SS in amoebae infected with the L. pneumophila wild-type strain JR32. Cryotomograms of L. pneumophila inside LCVs in either A. castellanii or D. discoideum at 2 h pi revealed that the Icm/Dot T4SS localizes to the bacterial cell poles also intracellularly (see Fig. S4 in the supplemental material) and that the bacterial outer membrane closely associates with the LCV membrane (distance <35 nm; *n*^events^ = 41). Taken together, the Icm/Dot T4SS localizes to the L. pneumophila poles extracellularly as well as intracellularly.

10.1128/mBio.02180-21.4 FIG S4 Icm/Dot T4SS localizes to the bacterial cell poles in infected amoebae. Download FIG S4, PDF file, 0.8 MB. Copyright © 2021 Böck et al. 2021 Böck et al. https://creativecommons.org/licenses/by/4.0/ This content is distributed under the terms of the Creative Commons Attribution 4.0 International license.

### Polar tethering of L. pneumophila correlates with Icm/Dot T4SS structure and function.

To further investigate a potential correlation between polar tethering and the structure and/or function of the Icm/Dot T4SS, we quantified cell pole tethering of L. pneumophila wild-type and different *icm* mutant strains (Δ*icmT*, Δ*icmE*, Δ*icmN*, and Δ*icmK* strains). The Icm/Dot machinery is composed of 27 different subunits, and complexes lacking individual subunits might still assemble but adopt distinct, impaired structures (12). Indeed, while the Δ*icmT*, Δ*icmE*, and Δ*icmN* mutants lack structural components of the T4SS but still form complexes (Fig. S3 and S5A in the supplemental material), the Δ*icmK* mutant (alias Δ*dotH* mutant) lacking the outer membrane component of the core transmembrane subcomplex does not assemble any T4SS complexes (36).

10.1128/mBio.02180-21.5 FIG S5 T4SS structure and activity influence cell-pole tethering at late infection stages. Download FIG S5, PDF file, 0.7 MB. Copyright © 2021 Böck et al. 2021 Böck et al. https://creativecommons.org/licenses/by/4.0/ This content is distributed under the terms of the Creative Commons Attribution 4.0 International license.

**FIG 2:**
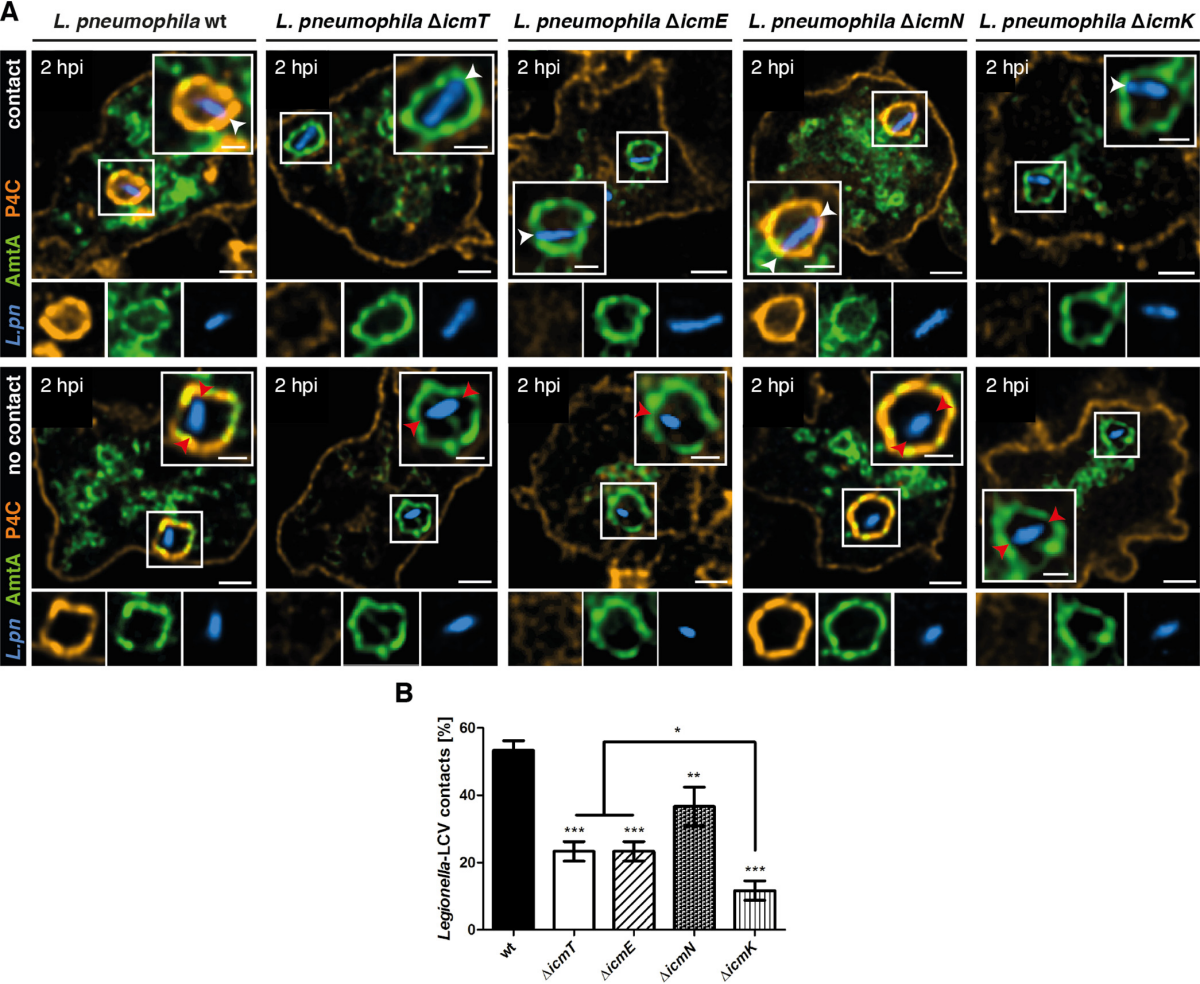
Polar tethering of L. pneumophila correlates with Icm/Dot T4SS structure and function. (A) Representative fluorescence micrographs of D. discoideum Ax3 producing P4C-mCherry (pWS032) and AmtA-GFP (pHK121) and infected (MOI of 5; 2 h) with mCerulean-producing L. pneumophila wild-type or Δ*icmT*, Δ*icmE*, Δ*icmN*, or Δ*icmK* mutant bacteria harboring plasmid pNP99. Examples are shown for contact between bacteria and the LCV membrane (top, white arrowheads) or no contact (bottom, red arrowheads). Scale bars, 2 μm (overview) and 1 μm (insert). (B) Quantification of data shown in panel A (*n*^events^ = 60). Data are represented as mean ± SD from three biological replicates (one-way ANOVA test; *, *P *< 0.05; **, *P *< 0.01; ***, *P *< 0.001).

In order to analyze LCV tethering of several *icm*/*dot* mutant strains, we switched from the labor-intensive cryoFIB milling/cryoET approach to CLSM, since fluorescence microscopy is more amenable to a higher sample throughput. In parallel, we also switched from A. castellanii to D. discoideum because only D. discoideum is genetically tractable, and for this amoeba many fluorescent probes are available. Accordingly, phosphatidylinositol 4-phosphate [PtdIns(4)*P*]-positive LCVs and phagosomes/endosomes can readily be visualized in D. discoideum. In order to visualize bacteria-containing vacuoles, D. discoideum dually labeled with the LCV/PtdIns(4)*P* probe P4C-mCherry (47) and the endosomal marker AmtA-green fluorescent protein (GFP) (48) were infected with L. pneumophila wild-type or *icm* mutant strains and analyzed by CLSM (Fig. 2A). Specific contact sites between the bacterial pole and the LCV membrane were quantified only for “expanded” LCVs (not for “tight” vacuoles, where the bacterium is firmly wrapped by the host membrane). Bacteria perpendicular to the focal plane were not considered.

Using this approach, more than 50% of wild-type L. pneumophila strains in LCVs were oriented with their poles in proximity to the vacuole membrane at 2 h pi (Fig. 2A and B; *n*^events^ = 60) and 8 h pi (Fig. S5B and C; *n*^events^ = 60), indicating that cell pole tethering might be required during various intracellular infection stages. Compared to the JR32 wild-type strain, we observed an approximately 2-fold reduction of cell pole tethering for the L. pneumophila Δ*icmT* strain at 2 h pi (Fig. 2A and B) and 8 h pi (Fig. S5B and C). The same reduction was observed for the Δ*icmE* strain (Fig. 2A and B). For the L. pneumophila Δ*icmK* strain, we observed a roughly 4-fold reduction (Fig. 2A and B), while the Δ*icmN* strain displayed only moderately lower levels of cell pole tethering, despite the lack of some structural components (Fig. 2A and B). Interestingly, LCVs harboring the Δ*icmN* strain accumulated the PtdIns(4)*P* probe P4C-mCherry, and thus, the mutant bacteria still produced a functional T4SS. This was not observed for the other *icm* mutant strains, which therefore produced a functionally impaired T4SS (Fig. 2A).

Overall, tethering was significantly lower for the Δ*icmT* and Δ*icmE* mutant strains, which produce incomplete T4SS complexes, indicating that structural T4SS components are required to establish tethering. The additional 2-fold reduction of tethering observed for the Δ*icmK* mutant, which lacks the entire T4SS structure, further supports this hypothesis. Since tethering was not completely abolished for the L. pneumophila Δ*icmK* strain, it seems likely that a fraction of cell poles might also associate randomly with the vacuole membrane. Nevertheless, the fact that tethering of the L. pneumophila Δ*icmN* strain coincided with increased T4SS activity suggests that the activity also contributes to cell pole tethering. In summary, CLSM confirmed our previous observations from cryoSEM and cryoET regarding polar tethering of L. pneumophila in LCVs (Fig. 1; see also Fig. S1 and S2). The fluorescence microscopy approach further indicated that cell pole tethering is promoted by both the presence and activity of the Icm/Dot T4SS.

### Dynamics of bipolar tethering of L. pneumophila to the LCV membrane are T4SS dependent.

**FIG 3:**
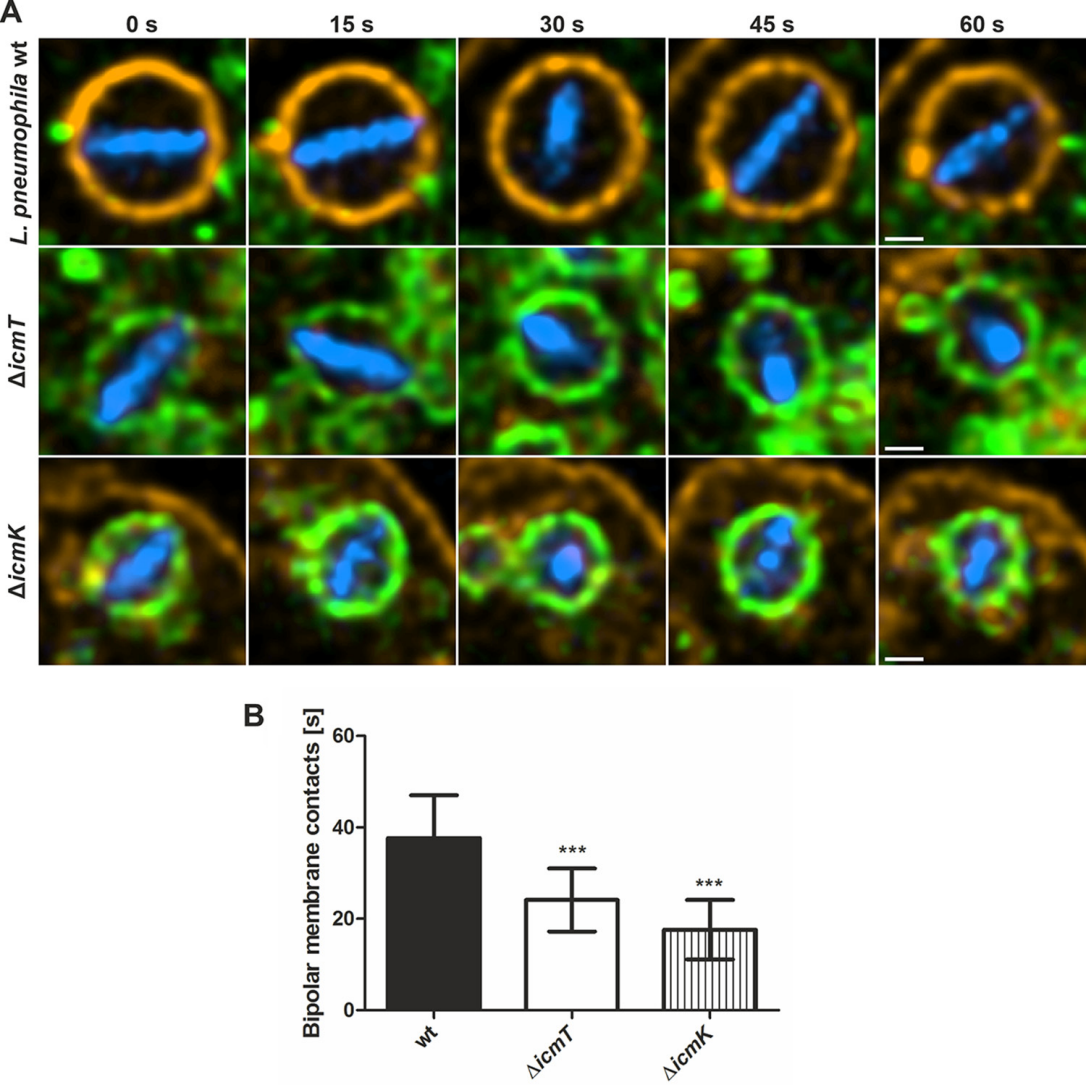
Dynamics of bipolar tethering of L. pneumophila to the LCV membrane are T4SS dependent. (A) Representative live-cell fluorescence micrographs of D. discoideum Ax3 producing P4C-mCherry (pWS032) and AmtA-GFP (pHK121) and infected (MOI of 5; 2 h) with mCerulean-producing L. pneumophila wild-type Δ*icmT* or Δ*icmK* mutant bacteria harboring plasmid pNP99. Infected cells were recorded for 60 s each. Scale bars, 0.5 μm. (B) Quantification of data shown in panel A (*n*^videos^ = 15). Data are represented as mean ± SD from two biological replicates (one-way ANOVA test; ***, *P *< 0.001).

In a complementary approach to correlate tethering of the L. pneumophila poles to the LCV membrane with the structure of the Icm/Dot T4SS, we used live-cell fluorescence microscopy. To this end, D. discoideum Ax3 producing P4C-mCherry and AmtA-GFP was infected for 2 h with mCerulean-producing L. pneumophila wild-type strain JR32, Δ*icmT*, or Δ*icmK* mutant bacteria, and the dynamics of the bacteria within their vacuoles were assessed for 60 s each (Fig. 3A; see also Movie S1 to S3 in the supplemental material). During this period of time, L. pneumophila wild-type, Δ*icmT*, or Δ*icmK* mutant bacteria made contact in a bipolar manner to the vacuole membrane for an average of ∼40 s, ∼25 s, or ∼20 s, respectively (Fig. 3B). Intriguingly, LCVs harboring the L. pneumophila wild-type strain tended to be larger (possibly promoting intravacuolar bacterial motility) and were decorated with PtdIns(4)*P*, while vacuoles harboring the Δ*icmT* or Δ*icmK* mutants were smaller (possibly restraining intravacuolar bacterial motility) and lacked PtdIns(4)*P*. Taken together, L. pneumophila wild-type bacteria make contact to the PtdIns(4)*P*-positive LCV membrane significantly longer than the Δ*icmT* or Δ*icmK* mutant bacteria residing in smaller, endosomal vacuoles. These results are in agreement with the notion that a structurally intact and fully functional T4SS promotes tethering to the pathogen vacuole membrane.

10.1128/mBio.02180-21.8 MOVIE S1 Live-cell imaging of D. discoideum Ax3 producing P4C-mCherry and AmtA-GFP and infected with the mCerulean-producing L. pneumophila wild-type. Download Movie S1, AVI file, 2.3 MB. Copyright © 2021 Böck et al. 2021 Böck et al. https://creativecommons.org/licenses/by/4.0/ This content is distributed under the terms of the Creative Commons Attribution 4.0 International license.

10.1128/mBio.02180-21.9 MOVIE S2 Live-cell imaging of D. discoideum Ax3 producing P4C-mCherry and AmtA-GFP and infected with the mCerulean-producing L. pneumophila Δ*icmT* mutant strain. Download Movie S2, AVI file, 3.7 MB. Copyright © 2021 Böck et al. 2021 Böck et al. https://creativecommons.org/licenses/by/4.0/ This content is distributed under the terms of the Creative Commons Attribution 4.0 International license.

10.1128/mBio.02180-21.10 MOVIE S3 Live-cell imaging of D. discoideum Ax3 producing P4C-mCherry and AmtA-GFP and infected with the mCerulean-producing L. pneumophila Δ*icmK* mutant strain. Download Movie S3, AVI file, 2.3 MB. Copyright © 2021 Böck et al. 2021 Böck et al. https://creativecommons.org/licenses/by/4.0/ This content is distributed under the terms of the Creative Commons Attribution 4.0 International license.

### L. pneumophila causes an indentation in the LCV membrane juxtaposed to a polar T4SS.

**FIG 4:**
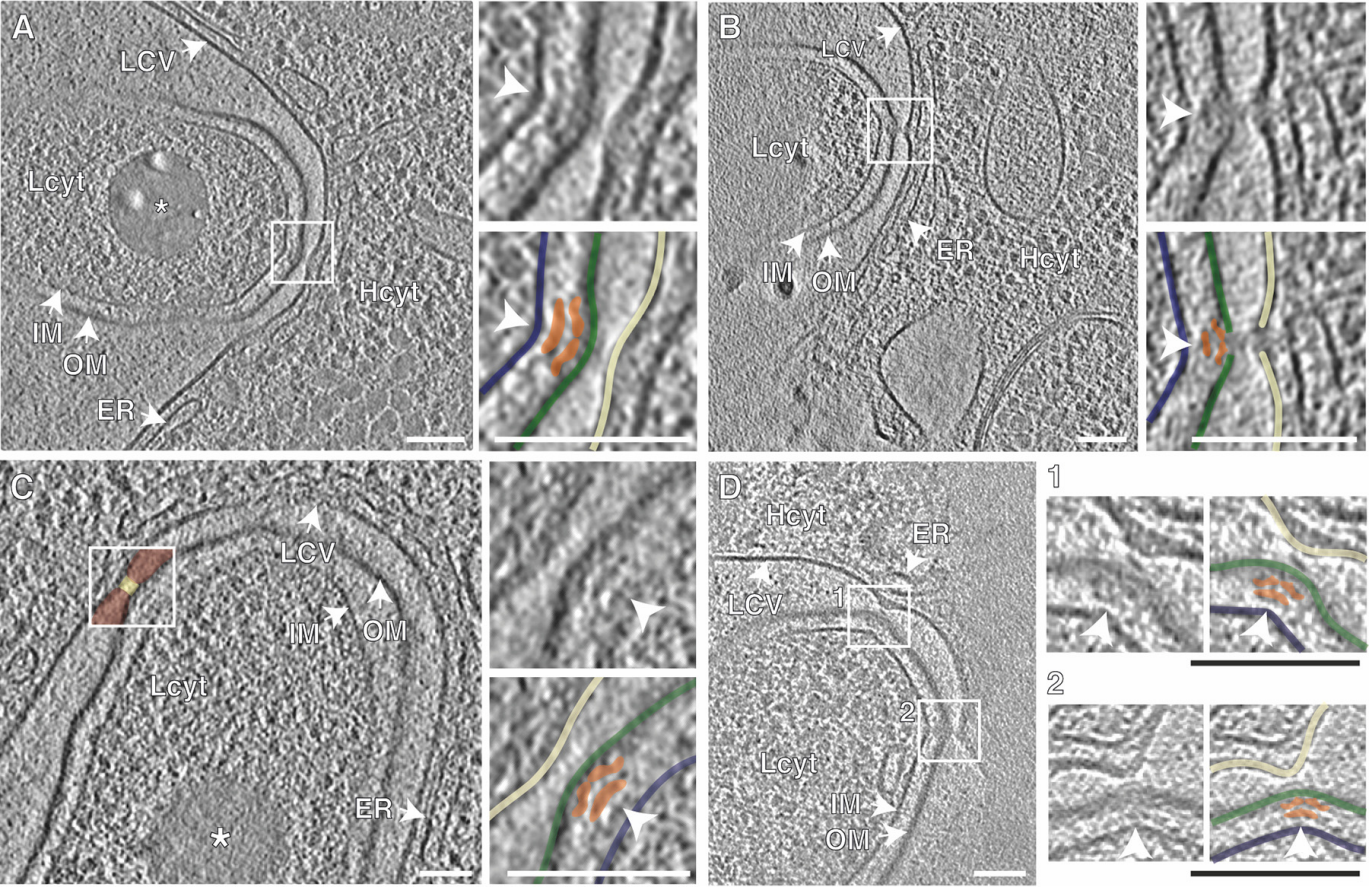
The polar L. pneumophila T4SS causes an indentation in the LCV membrane. (A to D) Representative interaction sites (*n* = 12) between the bacterial outer membrane and the indented LCV membrane localizing specifically to the site of the Icm/Dot T4SSs (white arrowheads) in D. discoideum (A, C) and A. castellanii (B, D). The observed interactions reduced the distance between the bacterial outer membrane and the LCV membrane (C, yellow-/brown-colored areas). (D) Multiple interaction sites can be present at one cell pole (*n* = 3). The images are representative of three biological replicates, where a total of 7 (D. discoideum) or 5 (A. castellanii) T4SS interaction sites were assessed. Shown are 12-nm tomographic slices of cryoFIB-processed lamellae and an overlay highlighting individual membranes and the T4SSs. OM/green, outer membrane; IM/blue, inner membrane; LCV/off-white, LCV membrane; white arrowhead/orange, T4SSs; Lcyt, L. pneumophila cytoplasm; Hcyt, host cell cytoplasm; ER, endoplasmic reticulum; asterisk, storage granule; scale bars (overviews, inserts), 100 nm.

Next, we analyzed in detail the architecture of the interactions between bacterial cell poles and the LCV membrane in infected A. castellanii and D. discoideum amoebae. As a general pattern, the bacteria localized either close to (<35 nm) or at quite a large distance (>300 nm) from the LCV membrane (*n* = 120). Strikingly, when the bacterial cell pole was within ∼35 nm reach of the LCV membrane at 2 h pi (*n* = 41), ∼30% of the observed T4SSs appeared to form distinct interaction sites, characterized by indentations in the LCV membrane juxtaposed to the precise site of a T4SS at the bacterial pole (Fig. 4A to C; *n*^contact sites^ = 12). We also identified multiple T4SS-LCV contact sites at the same bacterial pole (Fig. 4D; *n* = 3). We did not observe similar interaction sites between bacterial cell poles and the proximal LCV membranes in the absence of a T4SS. Importantly, membrane indentations with similar spacing and curvature were also not identified when the bacterial poles were at a greater distance to the LCV membrane. Finally, similar interaction sites were not observed for the L. pneumophila Δ*icmT* strain (Fig. S1D to E).

The distance between the bacterial outer membrane and the LCV membrane at these contact sites was on average ∼16 nm (Fig. 4; quantification area indicated in Fig. 4C as yellow area inside the box) compared to ∼31 nm in close proximity to these contact sites (quantification area indicated in Fig. 4C as brown area inside the box). Overall, data collected from infection experiments using two different amoeba hosts did not indicate the presence of an extended potential T4SS pilus that would mediate effector translocation. Hence, effector translocation might occur either through a short (few-nm-long) pilus-like structure or through direct contact between the bacterial outer membrane and the LCV membrane, mediated by distal parts of the T4SS complex. Interestingly, at one L. pneumophila-LCV contact site, the bacterial outer membrane and the LCV membrane appeared somewhat “smeared,” in agreement with the notion of membrane alterations at the contact site (Fig. 4B). In summary, our *in situ* data of L. pneumophila inside LCVs show tethering of bacterial cell poles to the LCV membrane during vacuole expansion and distinct contact sites (“indentations”) between tethered bacterial poles and the LCV membrane, specifically where a functional T4SS is present.

### LPS layers at Icm/Dot sites and L. pneumophila poles tethered to host membranes.

To allow for secretion of effector proteins across the host cell plasma membrane and/or the pathogen vacuole membrane, the pilus/conduit of a secretion system has to be long enough to cross the lipopolysaccharide (LPS) layer of the Gram-negative donor bacterium and establish contact with the target membrane. For instance, the length of the T3SS needle is actively regulated by a tape measure protein in various pathogens (e.g., *Yersinia*, *Shigella*, and Salmonella) to ensure that contact with the target membrane can be established (49–54). As a result, Salmonella enterica serovar Typhimurium (*S*. Typhimurium) with longer LPS (ranging from 35 to more than 100 O-antigen repeat units) is severely impaired for invasion, as the T3SS needle cannot contact the host cell membrane anymore (50).

We sought to study by cryoFIB milling and cryoEM the bacterial LPS layer at Icm/Dot sites and the L. pneumophila poles tethered to host membranes. To this end, we used human HeLa cells, which are better suited than amoebae for an analysis of early events during pathogen-host cell encounters. Specifically, we chose HeLa cells as a model, since their thin edges are easily accessible for cryoET imaging (55), and they have been shown to be more robust against high bacterial loads (56) than macrophages (57, 58). HeLa cells are infected by L. pneumophila rather inefficiently, and some bacteria remain adherent to the cell surface (59); this allows to frequently capture and visualize bacteria in close association with the plasma membrane.

**FIG 5:**
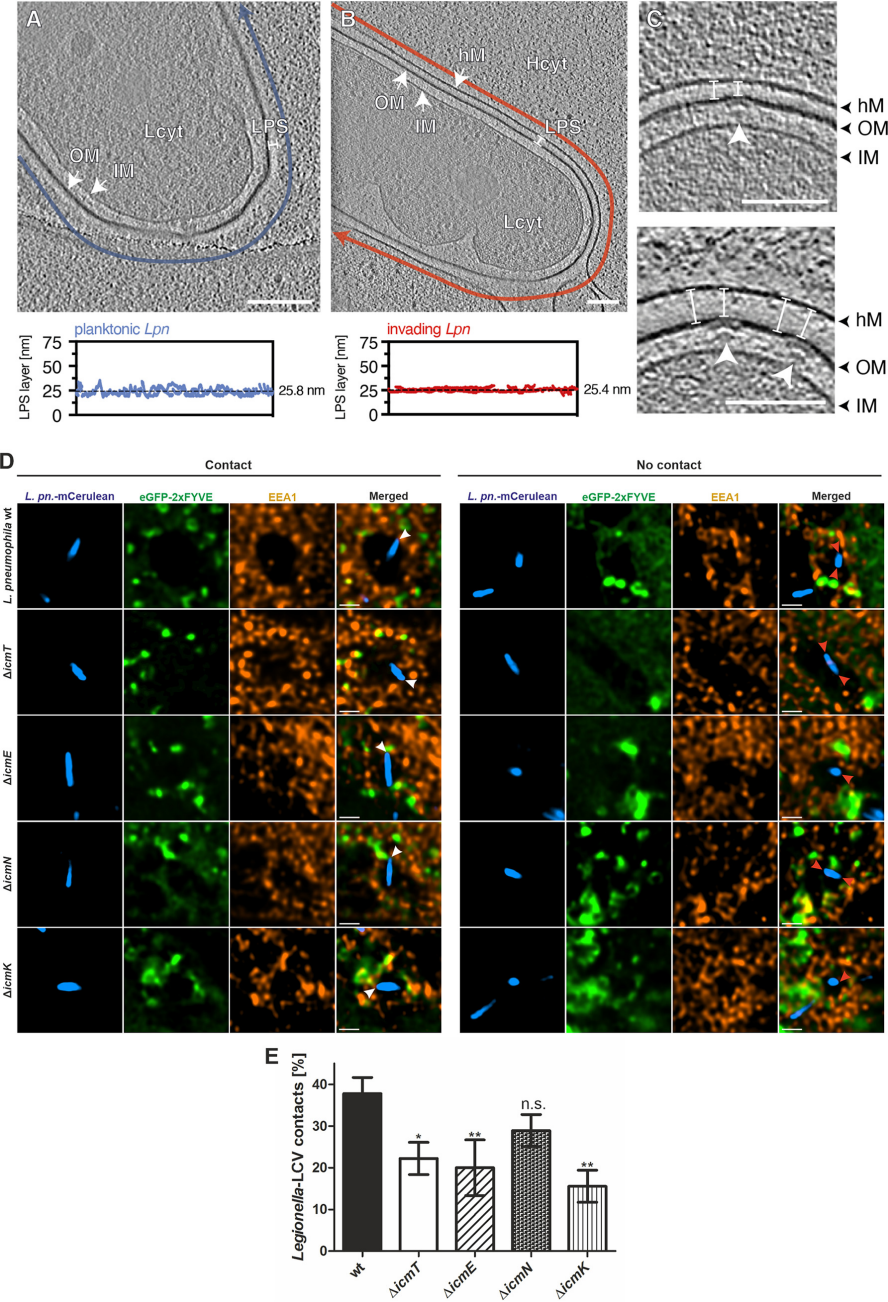
LPS layers at Icm/Dot sites and L. pneumophila poles tethered to host membranes. L. pneumophila LPS layer (A, B) or Icm/Dot T4SS (C) in planktonic bacteria (A) or infected (MOI of 75, 5 min pi) HeLa cells (B, C). The LPS layer thickness in planktonic L. pneumophila (∼26 nm; blue) is comparable to the gap between the bacterium and the HeLa cell (∼25 nm; red), suggesting that LPS serves as a physical barrier during host cell invasion. The distance between the bacterial outer membrane and the host cell membrane was constant along the bacterial cell body, indicated by a low standard deviation (B, bottom; approximately ±2 nm). Planktonic and adherent L. pneumophila show similar LPS layer thickness. (C) At T4SS assembly sites, the gap between the bacterium and the HeLa cell is slightly reduced (∼19 nm versus ∼25 nm, white arrowhead). Shown are 8-nm tomographic slices of cryotomograms. One tomogram was used for the quantification of the LPS layer as a representative of the population. Blue and red arrows indicate the path along which the thickness of the LPS layer was quantified. OM, outer membrane; IM, inner membrane; hM, host cell membrane; Lcyt, L. pneumophila cytoplasm; Hcyt, host cell cytoplasm; LPS, lipopolysaccharide; white arrowhead, T4SS; Scale bars, 100 nm. (D) Representative fluorescence micrographs of HeLa cells producing eGFP-2×FYVE (pEGFP-2×FYVE) and infected (MOI of 150; 1 h) with mCerulean-producing L. pneumophila wild-type or Δ*icmT*, Δ*icmE*, Δ*icmN*, or Δ*icmK* mutant bacteria harboring plasmid pNP99. Infected cells were fixed with PFA and stained with an anti-early endosome antigen 1 (EEA1) antibody prior to imaging. Examples are shown for contact between bacteria and the LCV membrane (left, white arrowheads) or no contact (right, red arrowheads). Scale bars, 1 μm. (E) Quantification of data shown in panel D (*n*^events^ = 45). Data are represented as mean ± SD from three biological replicates (one-way ANOVA test; *, *P *< 0.05; **, *P *< 0.01; n.s., not significant).

To estimate the minimal required length of a potential T4SS pilus/conduit, we quantified the thickness of the LPS layer in cryotomograms of L. pneumophila. To this end, we segmented cellular boundaries in cryotomograms, converted them to binary masks, and computed the minimal distance between the two masks as an estimate for the thickness of the LPS layer (see Fig. S6 in the supplemental material). Cryotomograms of L. pneumophila showed a contiguous electron-transparent layer around the bacterial outer membrane (Fig. 5A). The observed layer exhibited a reduction in density compared to that of the surrounding amorphous ice. Based on the thickness of this layer and previous reports from other bacterial species (60–62), we hypothesized that regions with reduced density around L. pneumophila reflect the presence of LPS.

10.1128/mBio.02180-21.6 FIG S6 Outline of the computational steps used for quantifying the thickness of LPS layers. Download FIG S6, PDF file, 0.4 MB. Copyright © 2021 Böck et al. 2021 Böck et al. https://creativecommons.org/licenses/by/4.0/ This content is distributed under the terms of the Creative Commons Attribution 4.0 International license.

Next, we analyzed the thickness of LPS layers during host cell entry. Using the quantification approach described above (Fig. S6), we found that LPS layers of planktonic L. pneumophila and bacteria adhering to HeLa cells (labeled as “invading”) displayed an average thickness of 25.5 nm ± 2.2 nm (Fig. 5A and B), indicating that LPS forms a constant physical barrier between the bacterium and the host cell. Taken together, planktonic and adherent L. pneumophila strains show a similar LPS layer thickness.

Intriguingly, at the assembly sites of T4SSs, the LPS layer of L. pneumophila entering HeLa cells seemed to be slightly thinner and spanned 19.2 nm ± 1.6 nm (Fig. 5C; 5 min pi; *n*^T4SSs^ = 11). If the LPS were of the same length at these sites, we would expect the LPS to push into the HeLa plasma membrane and create a small deformation, corresponding to the bulge in the bacterial outer membrane caused by a T4SS. However, such irregularities were not observed at the host cell plasma membrane, indicating that LPS can potentially be “squeezed” (or assembles differently) to reduce the distance to the host membrane. Alternatively, T4SSs could preferably assemble at bacterial pole sites with slightly shorter LPS to facilitate the interaction between the T4SS and the LCV membrane. Importantly, the thinner LPS layer (∼19 nm) correlates with our previous observation of ∼16-nm gaps at contact sites between the bacterial outer membrane and the LCV membrane (Fig. 4) and possibly allows for closer interactions between the bacterium and the host cell. In summary, the quantification of L. pneumophila LPS thickness revealed that the LPS layer is similar for planktonic and adherent/invading bacteria (∼26 nm), but slightly thinner at assembly sites of T4SSs (∼19 nm), corresponding approximately to the gap between the bacterial outer membrane and the LCV membrane at the contact sites. These observations are in agreement with the notion that LPS adopts a specific structure at these sites to accommodate a functional T4SS.

To validate our quantification procedure, we performed control experiments with *S.* Typhimurium. Cryotomograms of planktonic *S.* Typhimurium revealed that LPS forms a layer of constant thickness around the bacterial cell body of ∼30 nm (see Fig. S7A in the supplemental material). The thickness of the LPS layer was comparable between planktonic and invading bacteria (Fig. S7B), similar to what we previously observed for L. pneumophila (Fig. 5A and B). These results suggest that LPS layer thickness is not regulated during invasion and predetermines the distance between the bacterial outer membrane and the host cell membrane. Previous studies have shown that *S.* Typhimurium adapts its LPS upon host cell invasion to evade immune recognition (51, 63, 64). Our results revealed that at 1 h pi, the thickness of the LPS layer of intracellular *S.* Typhimurium is indeed reduced by ∼5 nm (Fig. S7C), validating our approach to quantify the LPS layer in cryotomograms.

10.1128/mBio.02180-21.7 FIG S7 LPS represents a physical barrier between bacteria and their environment. Download FIG S7, PDF file, 0.9 MB. Copyright © 2021 Böck et al. 2021 Böck et al. https://creativecommons.org/licenses/by/4.0/ This content is distributed under the terms of the Creative Commons Attribution 4.0 International license.

As an additional control, we quantified the thickness of the lipooligosaccharide (LOS) layer in Campylobacter jejuni. LOS is a low molecular weight form of LPS and consists of a lipid A that is linked to a polysaccharide but lacks the O-specific polysaccharide chain commonly found in other Gram-negative bacteria (65). Indeed, we found that the LOS layer of intracellular C. jejuni was significantly shorter (∼15 nm) than the LPS layer in *S.* Typhimurium (∼25 nm), further validating the accuracy of our LPS quantification protocols (Fig. S7D). Taken together, the LPS of planktonic or invading S. Typhimurium is of similar thickness (∼30 nm) and thinner than LPS or LOS of intracellular *S*. Typhimurium or *C. jejuni*, respectively.

Finally, we sought to validate the HeLa cells as a model for Icm/Dot-dependent polar tethering of L. pneumophila to the LCV membrane. To this end, HeLa cells producing the PtdIns(3)*P* probe eGFP-2×FYVE were infected with mCerulean-producing L. pneumophila wild-type or Δ*icmT*, Δ*icmE*, Δ*icmN*, and Δ*icmK* mutant bacteria, and immunostained for the early endosome antigen 1 (EEA1). Both endosomal markers localize to vacuoles harboring L. pneumophila only early during infection, but allow visualization of the vacuoles (Fig. 5D). Quantification of bacterial tethering to the vacuole membrane revealed that the contacts of the Δ*icmT*, Δ*icmE*, and Δ*icmK* mutant bacteria were approximately 2-fold less frequent, compared to those of the parental strain (Fig. 5E; *n*^events^ = 45). These findings are very similar to what we observed in amoebae (Fig. 2). In summary, HeLa cells infected with L. pneumophila are a valid model to assess the Icm/Dot-dependent polar tethering of the pathogen to the LCV membrane.

### Conclusions.

**FIG 6:**
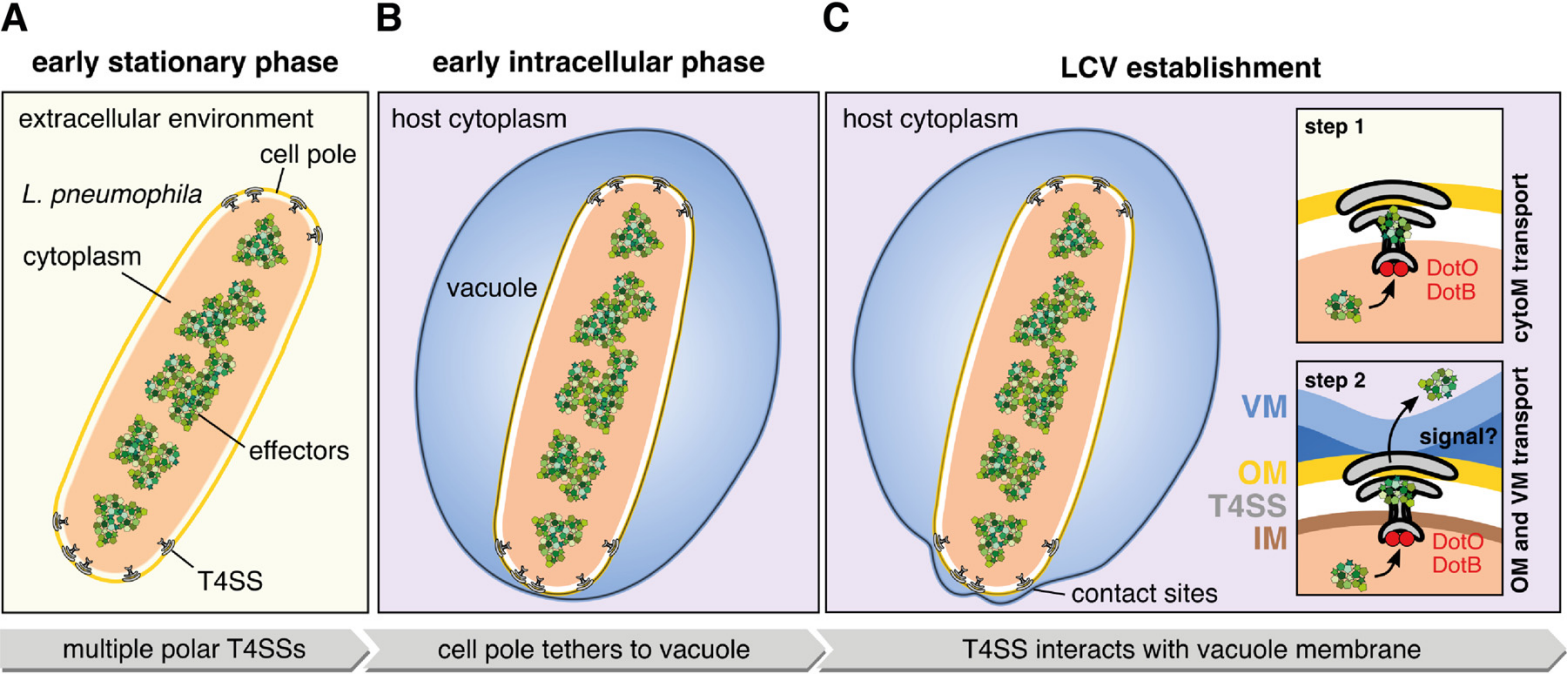
Potential mode of delivery of L. pneumophila T4SS effector proteins. (A) L. pneumophila assembles multiple T4SSs at the cell poles when grown to early stationary phase in AYE medium. (B) Once taken up by an amoeba host, the bacterium resides inside a vacuole and tethers its cell pole to the vacuole membrane to bring the T4SS in close proximity to the membrane. (C) Multiple T4SSs can establish contact with the vacuole membrane. Conformational changes in the cytoplasmic complex, triggered by the ATPases DotO and DotB (18), might activate the T4SS for effector transport across the cytoplasmic membrane (“cytoM transport”; step 1) by creating a channel within the cytoplasmic complex. These conformational changes can already be observed in L. pneumophila grown to stationary phase (A). Various, yet unknown, signals could further activate the T4SS to transport substrates across the bacterial outer and vacuole membrane (step 2). LCV, *Legionella*-containing vacuole; cytoM, cytoplasmic membrane; VM, vacuole membrane; OM, outer membrane; IM, inner membrane.

In conclusion, our *in situ* data show that the L. pneumophila wild-type and Δ*icmT* strain harbor polar Icm/Dot T4SSs (Fig. 6A). Intracellular L. pneumophila wild-type tethers its cell pole more frequently to the LCV membrane (Fig. 6B) compared to the Δ*icmT*, Δ*icmE*, Δ*icmN*, and Δ*icmK* mutant bacteria, suggesting that both T4SS structure and activity are required to establish tethering. Tethering of L. pneumophila wild-type bacteria establishes distinct contact sites characterized by indentations in the LCV membrane toward the T4SS (Fig. 6C). We hypothesize that the T4SS might tether the L. pneumophila cell pole to the LCV during vacuole expansion. Tethering brings the T4SS in close proximity of the LCV membrane (Fig. 6B), thereby facilitating subsequent effector translocation. The importance of tethering for intracellular survival and proliferation of L. pneumophila as well as the signals and/or T4SS structural components triggering tethering and effector translocation are yet to be discovered (Fig. 6C). Our study provides first insights into the interactions between the Icm/Dot T4SS and the LCV membrane in the *in vivo* context and suggests that translocation of effector proteins might be achieved either by close membrane contact independent of an extended pilus or by only a short (<20 nm) pilus/conduit.

## MATERIALS AND METHODS

### Bacteria, cells, and growth conditions.

Bacterial strains and cell lines used in this study are listed in Table 1. L. pneumophila strains were grown for 2 to 3 days on charcoal yeast extract (CYE) agar plates (66), buffered with *N*-(2-acetamido)-2-aminoethane sulfonic acid (ACES) at 37°C. Liquid cultures in ACES yeast extract (AYE) medium (67) were inoculated at an optical density at 600 nm (OD_600_) of 0.1 and grown at 37°C for 21 h to an early stationary phase (2 × 10^9^ bacteria/ml). Chloramphenicol (Cam) (5 μg/ml) was added for plasmid retention. S. enterica Typhimurium SL1344 (wild type) was grown in LB broth supplemented with 0.3 M NaCl and 50 μg/ml streptomycin (Str) (AppliChem) for 12 h at 37°C. These cultures were subcultured (1:20) in the same medium and incubated for 4 h at 37°C. C. jejuni strain 108 was grown in heart infusion (HI) broth (Sigma) at 160 rpm or on blood agar (BA) plates (BA base no. 2; Sigma) supplemented with Campylobacter selective supplements and defibrinated horse blood (Oxoid; lysed with 5% saponin). Cultures were grown under microaerophilic conditions (CampyGen sachet; Oxoid) in anaerobic jars.

**TABLE 1:** Strains and plasmids used in this study

Strain/plasmid	Relevant properties^*a*^	Reference or source
Strain		
A. castellanii		
5a2		ATCC PRA-228
D. discoideum		
Ax3		75
E. coli		
TOP10		Invitrogen
L. pneumophila		
GS3007 (Δ*icmN*)	L. pneumophila JR32 *icmN3007*::Kan^r^ (*lphA*)	10
GS3010 (Δ*icmK*)	L. pneumophila JR32 *icmK3010*::Kan^r^	10
GS3011 (Δ*icmT*)	L. pneumophila JR32 *icmT3011*::Kan^r^	45
JR32	Derivative of wild-type L. pneumophila strain Philadelphia-1 (serogroup 1)	76
LELA4432 (Δ*icmE*)	L. pneumophila JR32 *icmE*::Tn*903*dII*lacZ*	76
*S.* Typhimurium		
SL13144	Virulent SB300 wild-type strain	77
C. jejuni		
108	Virulent patient isolate	78
Plasmid		
pDM323	*Dictyostelium* extrachromosomal expression vector, C-terminal GFP, G418^r^	79
pDM1044	*Dictyostelium* extrachromosomal expression vector, C-terminal mCherry, Hyg^r^	80
peGFP-2×FYVE	Mammalian expression vector, fluorescent PtdIns(3)*P* probe	81
pHK121	pDM323-*amtA*-*gfp*	48
pNP99	L. pneumophila extrachromosomal expression vector, mCerulean, Cam^r^	47
pWS032	pDM1044-*P4C_SidC_*-*mCherry*	47

a Cam, chloramphenicol; Hyg, hygromycin; Kan, kanamycin; G418, Geneticin.

A. castellanii strain 5a2 (ATCC PRA-228) and D. discoideum strain Ax3 were grown as adherent cultures at 27°C and 23°C, respectively. A. castellanii was grown in Trypticase soy yeast (TSY) extract broth (30 g/liter TSY, 10 g/liter yeast extract; Sigma-Aldrich) and passaged every 5 days. D. discoideum Ax3 amoebae were cultivated in HL-5 medium (ForMedium) at 23°C in the dark. Cells were maintained every 2 to 3 days by rinsing with fresh HL-5 and by transferring 1 to 2% of the volume to a new T75 flask containing 10 ml of medium. Cells were strictly maintained below 90% confluence.

HeLa CCL-2 cells (human cervical adenocarcinoma; ATCC) were grown in Dulbecco modified Eagle medium (DMEM) (Gibco) supplemented with 10% heat-inactivated fetal calf serum (FCS) (BioConcept), 1% l-glutamine (Sigma), and 50 μg/ml Str at 37°C and 5% CO_2_ and passaged every 4 days. T84 cells (human colon carcinoma; Sigma) were grown in DMEM/F-12 (Sigma) supplemented with 5% FCS, nonessential amino acids (Sigma), and 1% l-glutamine (Sigma) at 37°C/5% CO_2_ and passaged every 4 days.

### Transformation of D. discoideum.

Transformation of axenic D. discoideum Ax3 amoeba was performed as described (44, 68). Briefly, D. discoideum was grown to approximately 80% confluence. The cells were collected in fresh HL-5 medium by centrifugation (450 × *g*, 5 min) and subsequently washed once with 5 ml Sorensen phosphate-buffer (SorC; 2 mM Na_2_HPO_4_, 15 mM KH_2_PO_4_, 50 μM CaCl_2_ × 2H_2_O, pH 6.0, autoclaved and stored at 4°C) and once with 5 ml electroporation buffer (EB) (10 mM KH_2_PO_4_, 50 mM sucrose, pH 6.1, filter-sterilized and stored at 4°C). The washing buffer was replaced with 2 ml fresh EB, and the cells were resuspended with a 5-ml serological pipette. Eight hundred microliters of the cell suspension each was added to a 4-mm gap electroporation cuvette (Bio-Rad). Two micrograms of both vectors were simultaneously added to the cuvette. Electroporation was performed with 2 pulses of 1 ms and 1 MV separated by a 5-s gap. Directly after electroporation, the cells were transferred into a T75 flask containing 10 ml HL-5. Around 24 h after electroporation, the medium was replaced with fresh HL-5, and the required selection antibiotics were added (20 μg/ml Geneticin, 50 μg/ml hygromycin). The medium was changed 72 h later. Upon obvious appearance of several microcolonies (usually 6 to 7 days after transformation), the cells were dislodged into fresh medium and transferred to a new flask.

### Infection assays for fluorescence microscopy.

D. discoideum producing the desired fluorescent probes were harvested from approximately 80% confluent cultures, seeded at 1 × 10^5^ cells/ml in 6-well plates (Corning) or 8-well μ-slides (for live-cell experiments) (ibidi) and allowed to adhere and grow for 24 h. Infections (multiplicity of infection [MOI] of 5) were performed with early-stationary-phase cultures of the L. pneumophila wild-type (JR32) and Δ*icmT*, Δ*icmE*, Δ*icmN*, or Δ*icmK* mutant strains harboring pNP99 (mCerulean), diluted in HL-5 and synchronized by centrifugation (450 × *g*, 10 min, room temperature [RT]) (69). Subsequently, infected cells were washed three times with HL-5 and incubated at 25°C for the time indicated. Finally, infected amoebae were recovered from the 6-well plates, fixed with 4% paraformaldehyde (PFA) for 30 min at room temperature, transferred to 8-well μ-slides, and embedded under a layer of phosphate-buffered saline (PBS)/0.5% agarose before imaging. For live-cell experiments, infected amoebae were directly imaged in the 8-well μ-slides after incubation.

HeLa CCL-2 cells were harvested from approximately 80% confluent cultures, seeded at 7.5 × 10^4^ cells/ml in 24-well plates (Corning) containing sterile coverslips and allowed to adhere for 24 h. Subsequently, cells were transfected with the desired fluorescent probe using the Lipofectamine 3000 kit (Thermo Fisher Scientific) according to the manufacturer’s protocol and incubated for a further 24 h at 37°C/5% CO_2_. Infections (MOI of 150) were performed with early stationary cultures of the L. pneumophila wild-type (JR32) and Δ*icmT*, Δ*icmE*, Δ*icmN*, or Δ*icmK* mutant strains harboring pNP99 (mCerulean), diluted in DMEM, and synchronized by centrifugation (450 × *g*, 10 min, RT). Subsequently, the infected cells were washed four times with DMEM and incubated at 37°C/5% CO_2_ for the time indicated. Next, HeLa cells were fixed with 4% PFA for 20 min at RT, permeabilized with PBS/0.25% Triton X-100 for 20 min, and blocked with PBS/1% bovine serum albumin (BSA) (1 h, RT). The cells were then incubated with a primary antibody against EEA1 (Abcam; ab2900), diluted 1:100 in blocking buffer for 2 h at RT, followed by an Alexa Fluor 594-coupled secondary antibody (Thermo Fisher Scientific; A21442) at a concentration of 1:200 (1 h, RT). Finally, the coverslips were washed three times with PBS and mounted on glass slides using ProLong Diamond antifade mounting medium (Thermo Fisher Scientific) and imaged.

### Confocal microscopy of infected cells.

Confocal microscopy of infected fixed or live cells was performed as described (23, 47, 69, 70) using a Leica TCS SP8 X CLSM with the following setup: white light laser, 442-nm diode, and HyD hybrid detectors used for each channel. Pictures were taken using a HC PL APO CS2 63×/1.4 oil objective with Leica Type F immersion oil and analyzed with Leica LAS X software. Settings for fluorescence imaging were as following: mCerulean (excitation, 442 nm; emission, 469 nm), eGFP (excitation, 488 nm; emission, 516 nm), mCherry (excitation, 568 nm; emission, 610 nm), and Alexa Fluor 594 (excitation, 590 nm; emission, 617 nm). Images and movies were captured with a pinhole of 1.19 Airy units (AU) and with a pixel/voxel size at or close to the instrument’s Nyquist criterion of approximately 43 × 43 × 130 nm (xyz). A scanning speed of 400 Hz, bidirectional scan, and line accumulation equal to 2 were used to capture still images (Fig. 2 and 5). Scanning speed of 700 Hz and bidirectional scan were used to capture movies (see Movies S1 to S3 in the supplemental material; Fig. 3).

### Image processing.

All images and movies were deconvolved with Huygens professional version 19.10 (Scientific Volume Imaging, The Netherlands; http://svi.nl) using the CMLE algorithm with 40 iterations and a 0.05 quality threshold. Signal to noise ratios were estimated from the photons counted for a given image. Single images, Z-stacks, and movies were finalized and exported with Imaris 9.5.0 software (Bitplane, Switzerland).

### Infection assays for electron microscopy.

For electron microscopy, amoebae or HeLa cells were grown directly on grids as previously described (41). Briefly, amoebae (5 × 10^5^ per well) or HeLa cells (3 × 10^4^ per well) were seeded onto EM gold finder grids (Au NH2 R2/2, Quantifoil) and incubated for 1 h (amoebae) or overnight (HeLa) to allow the cells to attach to the grids. Infected T84 cells (1 × 10^5^ per grid) were directly applied onto the grids (Cu R2/1, Quantifoil). Cells were infected at an MOI of 75 (L. pneumophila for HeLa and A. castellanii), 100 (L. pneumophila for D. discoideum; C. jejuni for T84), or 300 (*S.* Typhimurium for HeLa). Samples were vitrified at different infection time points (L. pneumophila, 5 min, 30 min, 2 h, and 3 h; *S.* Typhimurium, 20 min and 1 h; C. jejuni, 2 h).

### Vitrification of infected host cells.

Plunge-freezing was performed as described previously (41, 71). In short, gold finder grids (Au NH2 R2/2, Quantifoil) containing infected HeLa cells or amoebae or copper grids (Cu R2/1, Quantifoil) containing T84 cells were vitrified by back blotting (Teflon; 2× 3 to 7 s). Grids containing L. pneumophila cells were frozen on copper grids (Cu R2/2, Quantifoil). All grids were plunge-frozen in liquid ethane-propane (37%/63%) using a Vitrobot (Thermo Fisher) and stored in liquid nitrogen until further use.

### Cryo-focused ion beam milling.

Cryo-focused ion beam (cryoFIB) milling was used to prepare samples of plunge-frozen infected amoebae for imaging by cryo-electron tomography (cryoET) (72). Frozen grids with infected cells were processed as previously described (41) using a Helios NanoLab 600i dual beam FIB/SEM instrument (Thermo Fisher). Briefly, lamellae with ∼2 μm thickness were generated first (at 30 kV and ∼400 pA). The thickness of the lamellae (final, ∼200 nm) was then gradually reduced using decreasing ion beam currents (final, ∼25 pA). Lastly, lamellae were examined at low voltage by cryoSEM imaging (3 kV, ∼0.17 nA) to visualize intracellular bacteria. CryoFIB-processed grids were unloaded and stored in liquid nitrogen until further use.

### Cryo-electron microscopy and cryo-electron tomography.

Frozen grids were examined by cryo-electron microscopy (cryoEM) and cryo-electron tomography (cryoET) (71). Data were collected on a Titan Krios TEM (Thermo Fisher) equipped with a Quantum LS imaging filter and K2 Summit (Gatan). The microscope was operated at 300 kV, and the imaging filter was set to a slit width of 20 eV. The pixel size at the specimen level was ranging from 3.45 to 5.42 Å. Tilt series were recorded from −60° to +60° with 1° (whole bacterial cells) or 2° increments (lamellae) and −8-μm defocus. The total dose of a tilt series was 90 e^−^/Å^2^ (planktonic L. pneumophila), 150 e^−^/Å^2^ (planktonic *S.* Typhimurium), 100 e^−^/Å^2^ (invading L. pneumophila), 120 e^−^/Å^2^ (invading and intracellular *S.* Typhimurium), 75 e^−^/Å^2^ (intracellular L. pneumophila), or 110 e^−^/Å^2^ (intracellular C. jejuni). Tilt series and 2D projection images were acquired automatically using SerialEM. Three-dimensional reconstructions and segmentations were generated using the IMOD program suite (73).

### Quantification of lipopolysaccharide and lipooligosaccharide layers.

LPS and LOS layer quantification was done across the whole bacterial cell body, and accordingly, several hundreds of values were obtained for each condition. Cryotomograms were converted to TIFF images carrying the respective metadata. A representative 2D section was selected for segmentations of cellular boundaries (bacterium versus extracellular space; bacterium versus host cell) using the carving tool in ilastik (version 1.3.2 [74]) (see Fig. S6 in the supplemental material). Segmentations were exported as binary masks using ilastik. The masks were reduced to their edges (Canny edge detector) to compute the minimum distance from each pixel on one edge to the other edge. The gap between the two edges corresponds to the bacterial LPS or LOS layer. Data were processed and analyzed using Julia 1.0.3 and Images.jl 0.18.0 software.

### Statistical analyses.

Data from at least two independent infection experiments (for cryoET analysis) were analyzed by a two-tailed Student's *t* test to compare wild-type to mutant infections. Data from at least three (Fig. 2 and 5) or two (Fig. 3) independent infection experiments (fluorescence microscopy) were analyzed by one-way analysis of variance (ANOVA).

### Data availability.

We declare that all data sets generated during this study are available from the corresponding authors upon reasonable request. Key tomograms have been submitted to EMDB (https://deposit-pdbe.wwpdb.org/deposition) under accession numbers EMD-13246, EMD-13247, EMD-13248, and EMD-13249.
