# Supplementary material for: The Polar Legionella Icm/Dot T4SS Establishes Distinct Contact Sites with the Pathogen Vacuole Membrane
Source: mBio. 2021 Oct 12;12(5):e02180-21. doi: 10.1128/mBio.02180-21 (PMC8510526; doi:10.1128/mBio.02180-21)
Supplement: FIG S1 [file mbio.02180-21-sf001.pdf]

Supplementary Figures

Figure S1

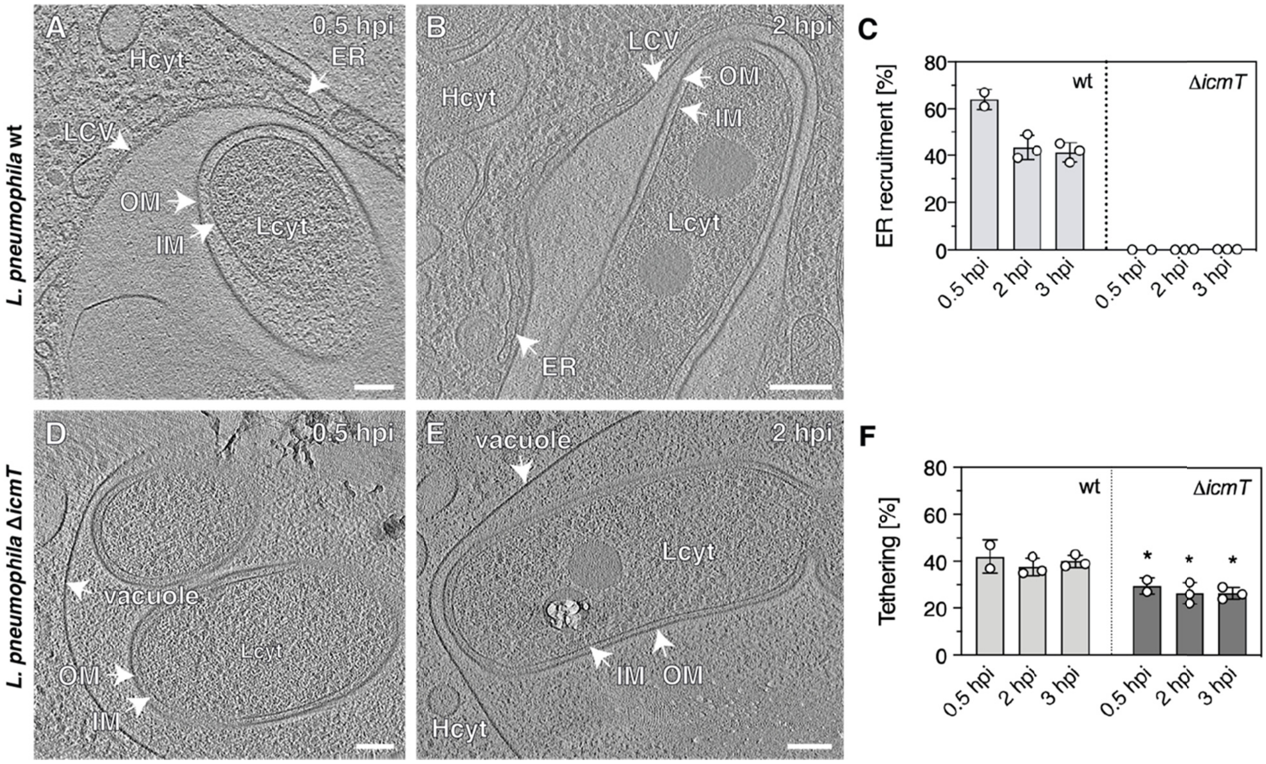

Figure S1. ***L. pneumophila*** **wild-type tethers its cell pole to the LCV membrane in** ***D. discoideum*****.** Representative 2-D images of cryotomograms of (**A-B**) *L. pneumophila* wild-type residing in a mature, ER-decorated LCV (30 min pi, n^{LCVs}=18; 2 hpi, n^{LCVs}=23). Vacuoles containing (**C-E**) Δ*icmT* mutant bacteria were never decorated with ER (30 min pi, n^{LCVs}=7; 2 hpi, n^{LCVs}=6). (**F**) Quantifications of cell-pole-tethering over time. Data in (**C**) and (**F**) are represented as mean ± SD. from at least two independent infection experiments *(*P<0.05*). Shown are 12 nm tomographic slices. OM, outer membrane; IM, inner membrane; LCV, LCV membrane; Lcyt, *L. pneumophila* cytoplasm; Hcyt, host cell cytoplasm; ER, endoplasmic reticulum; Scale bars, 100 nm.
