# Supplementary material for: The Polar Legionella Icm/Dot T4SS Establishes Distinct Contact Sites with the Pathogen Vacuole Membrane
Source: mBio. 2021 Oct 12;12(5):e02180-21. doi: 10.1128/mBio.02180-21 (PMC8510526; doi:10.1128/mBio.02180-21)
Supplement: FIG S2 [file mbio.02180-21-sf002.pdf]

Figure S2

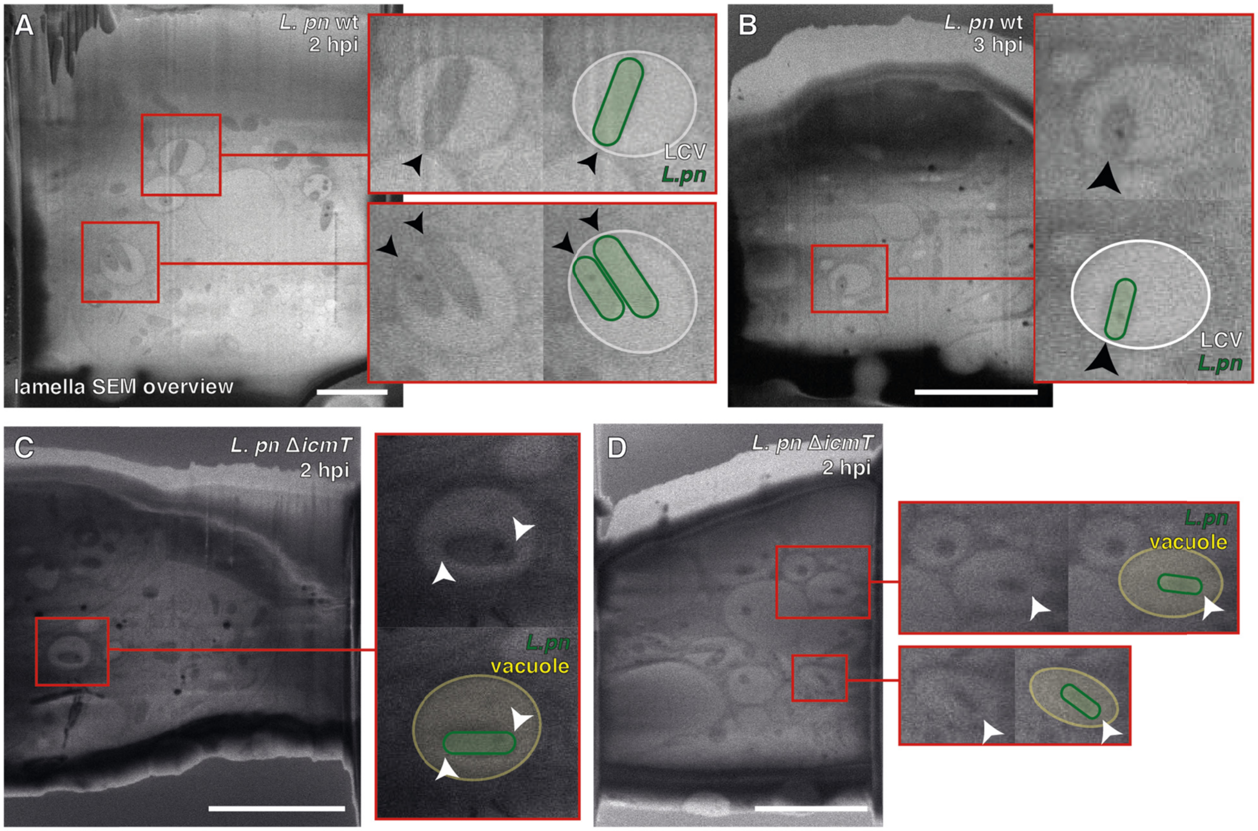

Figure S2. *L. pneumophila* wild-type tethers its cell poles to the LCV membrane. *A. castellanii* amoebae were infected (MOI 75) with *L. pneumophila* (**A, B**) wild-type or (**C, D**) Δ*icmT* mutant bacteria and analyzed by cryoSEM. Tethering of the cell poles (black arrowheads) of *L. pneumophila* wild-type bacteria (green) to the LCV membrane (white) at (**A**) 2 hpi (~49%; $n^{vacuoles}$=137) or (**B**) 3 hpi (~53%; $n^{vacuoles}$=86). Tethering is clearly visible in expanded LCVs, where single bacterial poles attach to the pathogen vacuole. (**C, D**) Tethering of cell poles (white arrowheads) of *L. pneumophila* Δ*icmT* mutant bacteria (green) to the vacuole membrane (yellow) was observed at lower frequency at 2 hpi (~30%, $n^{vacuoles}$=29). Scale bars, 5 µm.
