# Supplementary material for: The Polar Legionella Icm/Dot T4SS Establishes Distinct Contact Sites with the Pathogen Vacuole Membrane
Source: mBio. 2021 Oct 12;12(5):e02180-21. doi: 10.1128/mBio.02180-21 (PMC8510526; doi:10.1128/mBio.02180-21)
Supplement: FIG S3 [file mbio.02180-21-sf003.pdf]

Figure S3

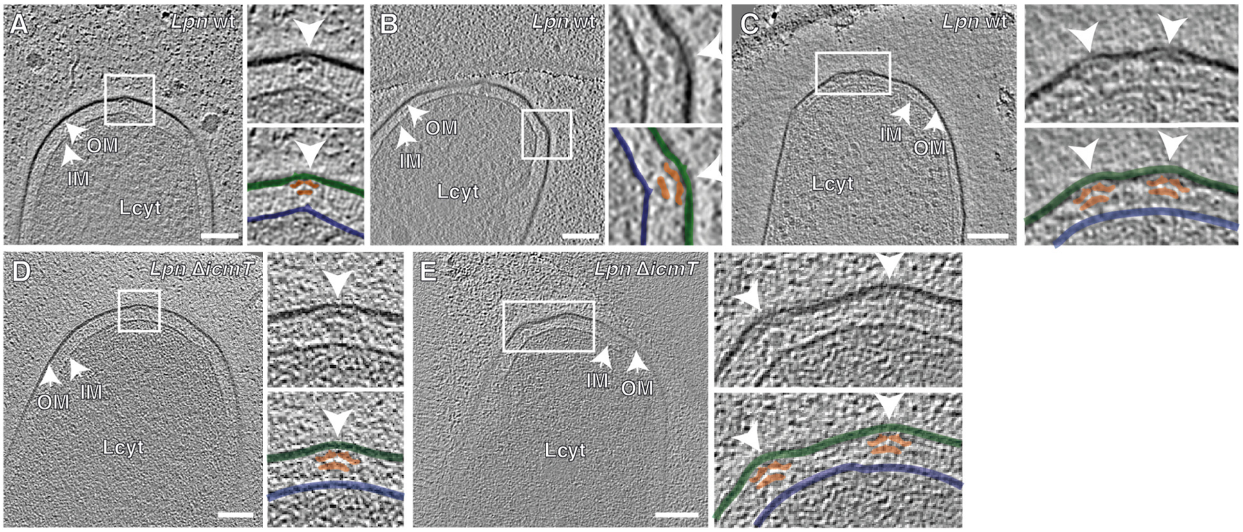

Figure S3. The *L. pneumophila* Icm/Dot T4SS is located at bacterial cell poles. Cryotomograms indicating that the Icm/Dot T4SSs (white arrowheads, orange) localized to the poles of *L. pneumophila* (**A-C**) wild-type as well as (**D, E**) Δ*icmT* mutant bacteria. Secretion systems in both strains adopted the characteristic "Wi-Fi"-like structure of the T4SS. Multiple secretion systems can be distributed along a cell pole. Shown are 8 nm slices of cryotomograms. OM/green, outer membrane; IM/blue, inner membrane; Lcyt, *L. pneumophila* cytoplasm; scale bars, 100 nm.
