# Supplementary material for: The Polar Legionella Icm/Dot T4SS Establishes Distinct Contact Sites with the Pathogen Vacuole Membrane
Source: mBio. 2021 Oct 12;12(5):e02180-21. doi: 10.1128/mBio.02180-21 (PMC8510526; doi:10.1128/mBio.02180-21)
Supplement: FIG S4 [file mbio.02180-21-sf004.pdf]

Figure S4

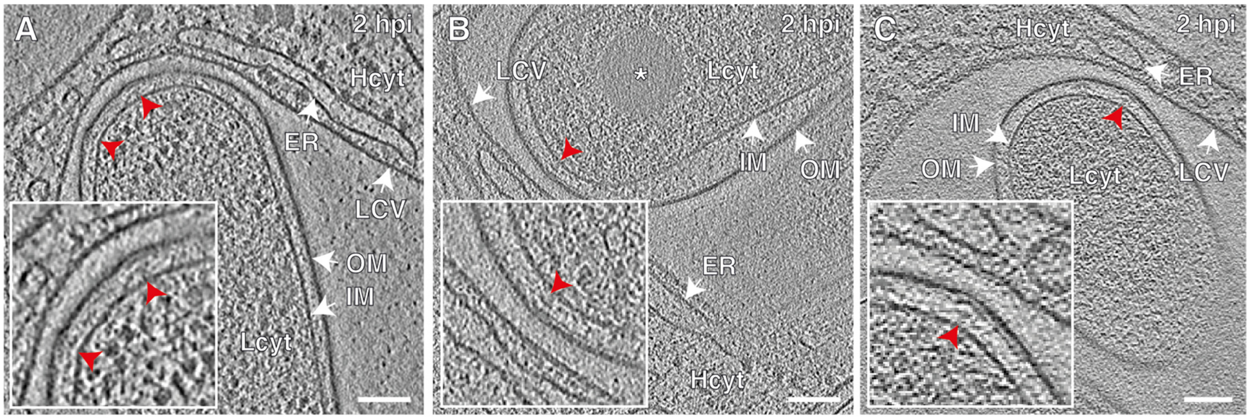

Figure S4. The Icm/Dot T4SS localizes to the bacterial cell poles in infected amoebae. Cryotomograms showing that intracellular *L. pneumophila* wild-type harbored one or more T4SSs at the bacterial cell pole (red arrowhead) in (**A**) *A. castellanii* and (**B, C**) *D. discoideum* at 2 hpi. Shown are 12 nm slices of cryoFIB-processed lamellae. OM, outer membrane; IM, inner membrane; LCV, LCV membrane; Lcyt, *L. pneumophila* cytoplasm; Hcyt, host cell cytoplasm; ER, endoplasmic reticulum; red arrowhead, T4SSs; asterisk, storage granule; scale bars, 100 nm.
