# Supplementary material for: The Polar Legionella Icm/Dot T4SS Establishes Distinct Contact Sites with the Pathogen Vacuole Membrane
Source: mBio. 2021 Oct 12;12(5):e02180-21. doi: 10.1128/mBio.02180-21 (PMC8510526; doi:10.1128/mBio.02180-21)
Supplement: FIG S5 [file mbio.02180-21-sf005.pdf]

Figure S5

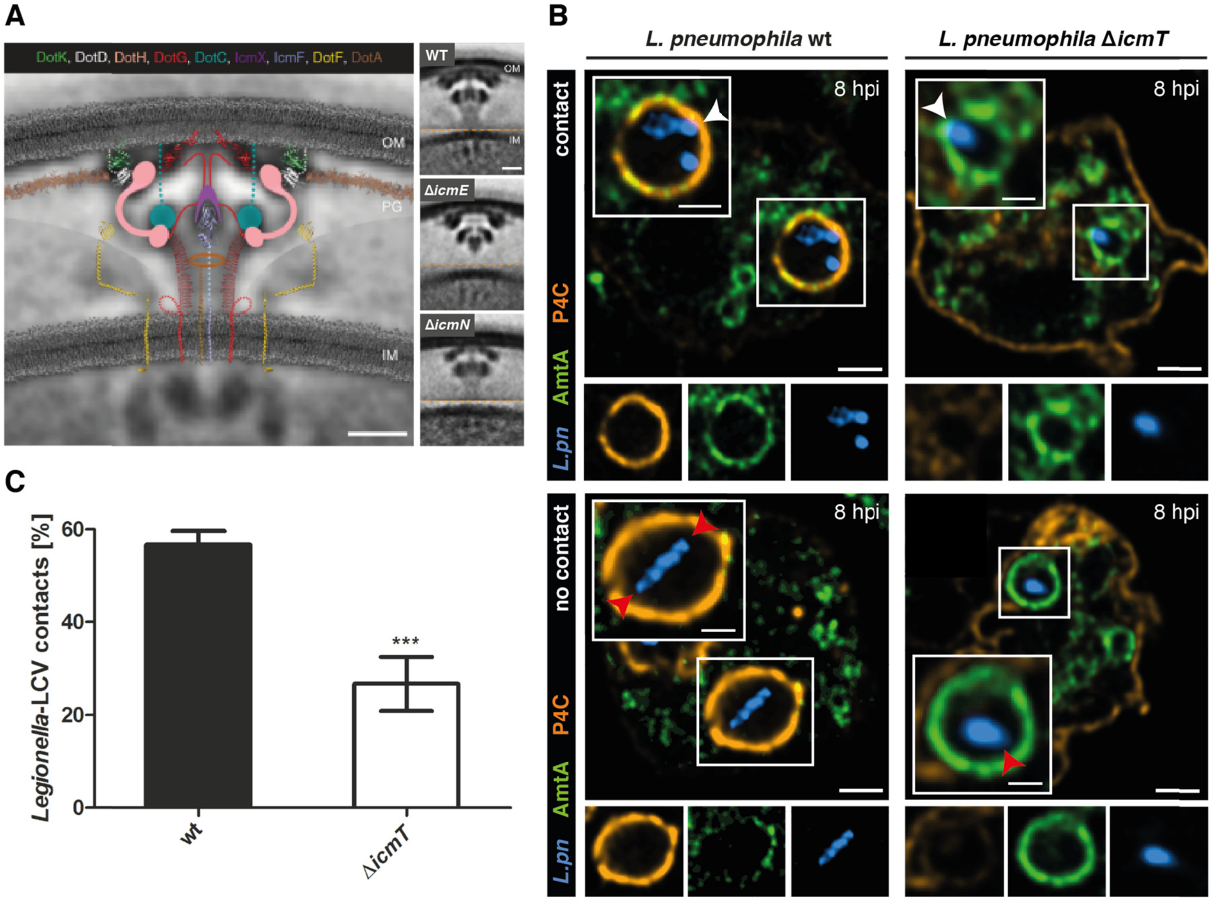

Figure S5. T4SS structure and activity influence cell-pole tethering at late infection stages. (**A**) Position of subunits of the T4SS and sub-tomogram averages of T4SS particles from *L. pneumophila* wild-type, Δ*icmE*, and Δ*icmN* mutant bacteria. Images taken from Ghosal *et al.* (2019) *Nat Microbiol 4*: 1173-1182. Scale bars, 10 nm. (**B**) Representative fluorescence micrographs of *D. discoideum* Ax3 producing P4C-mCherry (pWS032) and AmtA-GFP (pHK121), infected (MOI 5; 8 h) with mCerulean-producing *L. pneumophila* wild-type and Δ*icmT* mutant bacteria harboring plasmid pNP99. Examples are shown for contact between bacteria and the LCV membrane (top, white arrowheads) or no contact (bottom, red arrowheads). Scale bars: 2 µm (overviews), 1 µm (inserts). (**C**) Quantification of data shown in (B) (n^{events}=60). Data are represented as mean ± SD from three independent experiments (****P*<0.001).
