# Supplementary material for: The Polar Legionella Icm/Dot T4SS Establishes Distinct Contact Sites with the Pathogen Vacuole Membrane
Source: mBio. 2021 Oct 12;12(5):e02180-21. doi: 10.1128/mBio.02180-21 (PMC8510526; doi:10.1128/mBio.02180-21)
Supplement: FIG S6 [file mbio.02180-21-sf006.pdf]

Figure S6

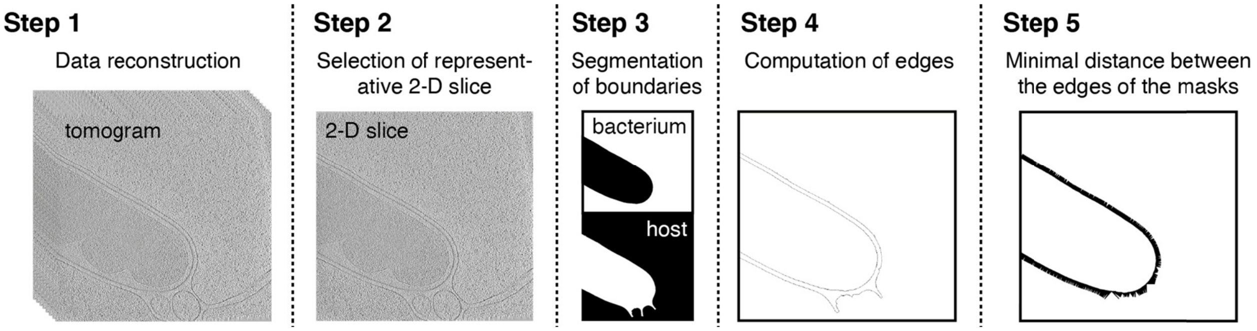

Figure S6. Outline of the computational steps used for quantifying the thickness of LPS layers. After reconstruction of cryoET data (step 1), tomograms were converted to TIFF images carrying the respective metadata. A representative 2-D slice was manually selected for segmentation of bacterial and eukaryotic cell boundaries (step 2). The bacterium and the host cell were segmented using the carving tool in ilastik ("the interactive learning and segmentation toolkit", Berg *et al*. (2019) *Nat Methods 16*: 1226-1232; step 3). Next, these boundary models were exported as binary masks, and the edges of each mask were computed (Canny edge detection; step 4). The distance between a pixel on the bacteria-mask to the closest pixel on the host-cell-mask was computed as a measure of LPS thickness (step 5). Data processing and analyses were performed using the software Julia 1.0.3 and Images.jl 0.18.0.
