# Supplementary material for: The Polar Legionella Icm/Dot T4SS Establishes Distinct Contact Sites with the Pathogen Vacuole Membrane
Source: mBio. 2021 Oct 12;12(5):e02180-21. doi: 10.1128/mBio.02180-21 (PMC8510526; doi:10.1128/mBio.02180-21)
Supplement: FIG S7 [file mbio.02180-21-sf007.pdf]

Figure S7

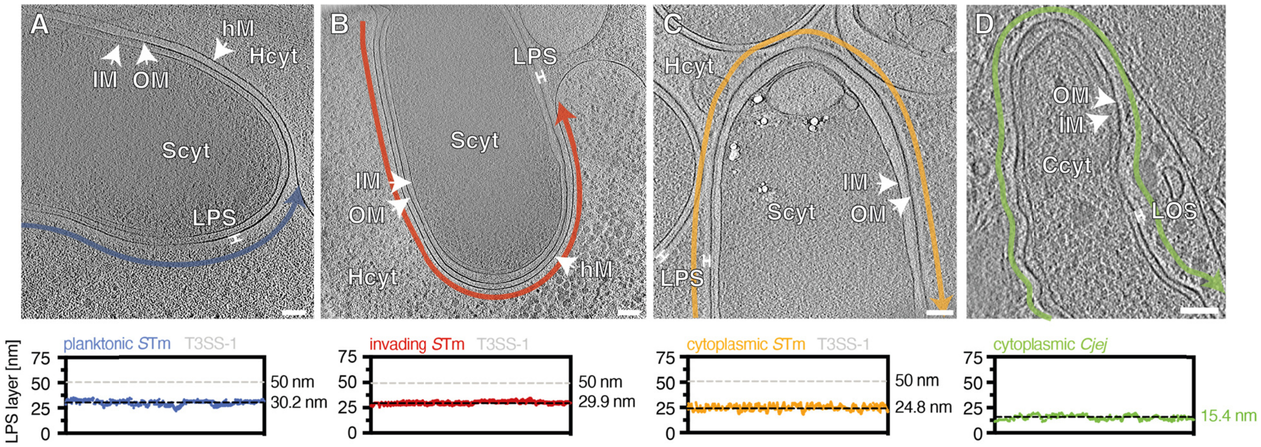

Figure S7. LPS represents a physical barrier between bacteria and their environment. (**A, B**) The LPS layer of (**A**) planktonic (~30 nm; blue) or (**B**) invading *S*. Typhimurium (~30 nm; red) is shorter than the recently reported length of its T3SS-1 needle (~50 nm, Park *et al*. (2018) *eLife 7*: e39514; indicated as grey line, lower panels), allowing the needle to contact the host cell membrane and secrete effector proteins into the host cell cytosol. (**C**) Intracellular *S*. Typhimurium adapts the length of its LPSs (~25 nm; yellow) in order to avoid immune recognition. (**D**) In comparison, LOS in *C. jejuni* was significantly shorter (~15 nm; green) than LPS of intracellular *L. pneumophila* (~25 nm; see Fig. 5) and *S*. Typhimurium (25-30 nm; A-C). Shown are (**A, B**) 8 nm (whole cells) and (**C, D**) 12 nm (cryoFIB-processed lamellae) tomographic slices of cryotomograms. One tomogram was used for the quantification of the LPS layer as a representative of the population. Blue, red, yellow and green arrows (upper panels) indicate the path along which the thickness of the LPS layer was quantified. OM, outer membrane; IM, inner membrane; hM, host cell membrane; Scyt, *S*. Typhimurium cytoplasm; Ccyt, *C. jejuni* cytoplasm; Hcyt, host cell cytoplasm; LPS, lipopolysaccharide; LOS, lipooligosaccharide; scale bars, 100 nm.
